# Construction and analysis of a lncRNA (*PWRN2*)-mediated ceRNA network reveal its potential roles in oocyte nuclear maturation of patients with PCOS

**DOI:** 10.1186/s12958-018-0392-4

**Published:** 2018-08-03

**Authors:** Xin Huang, Jiaping Pan, Bi Wu, Xiaoming Teng

**Affiliations:** Department of Assisted Reproductive Medicine, Shanghai First Maternity and Infant Hospital, Tongji University School of Medicine, 2699 Gaoke Road West, Shanghai, 200001 People’s Republic of China

**Keywords:** ceRNA, Oocyte maturation, Polycystic ovary syndrome, *PWRN2*, miR-92b-3p, *TMEM120B*

## Abstract

**Background:**

Polycystic ovary syndrome (PCOS) is a common endocrine and metabolic disorder in women. An lncRNA, namely, Prader-Willi region nonprotein coding RNA 2 (*PWRN2*), was up-regulated in the cumulus cells of patients with PCOS. However, the molecular mechanism of *PWRN2* in PCOS remains largely unknown.

**Methods:**

In this study, the expression levels of *PWRN2* were tested in cumulus cells through qRT-PCR analysis to confirm its potential roles in oocyte nuclear maturation of PCOS. A *PWRN2*-mediated ceRNA network was constructed based on three microarray datasets to investigate the molecular mechanism of *PWRN2* in oocyte development of patients with PCOS. The direct interactions of the candidate genes of the ceRNA network were also demonstrated by dual-luciferase reporter assay.

**Results:**

*PWRN2* was found to be associated with oocyte nuclear maturation in patients with PCOS in contrast to that in normal patients. Based on the microarray data, 176 lncRNAs (118 up-regulated and 58 down-regulated) and 131 mRNAs (84 up-regulated and 47 down-regulated) were identified to be regulated by *PWRN2*. A *PWRN2*-miR-92b-3p-*TMEM120B* ceRNA network was constructed based on results of analysis of the combined three microarray datasets (lncRNA+mRNA microarray in KGN/shPWRN2 in this study, miRNAs microarray and lncRNA+mRNA microarray in PCOS cumulus cells reported in previous studies). The coexpression characteristics of the genes (*PWRN2*, miR-92b-3p and *TMEM120B*) were detected in the cumulus cells of cumulus-oocyte complexes at different nuclear maturity stages in PCOS. These results are in accordance with the ceRNA hypothesis. Moreover, luciferase activity assay revealed that miR-92b-3p directly binds to *PWRN2* and targets *TMEM120B*.

**Conclusions:**

*PWNR2* plays important roles in oocyte nuclear maturation in PCOS by functioning as a ceRNA to reduce the availability of miR-92b-3p for *TMEM120B* target binding during oocyte maturation in PCOS. Our findings would provide new information and clarify abnormal oocyte development in PCOS.

**Electronic supplementary material:**

The online version of this article (10.1186/s12958-018-0392-4) contains supplementary material, which is available to authorized users.

## Background

Polycystic ovary syndrome (PCOS) is a common endocrinopathy in women of reproductive age [1] and accounts for approximately 75% of anovulatory infertility disorders [2]. The phenotype of PCOS is variable and includes hyperandrogenism, menstrual irregularity and polycystic ovarian morphology [3]. Patients suffering from PCOS are often diagnosed with obesity, hirsutism, insulin resistance, increased risk of endometrial cancer, metabolic syndrome [4], type 2 diabetes (T2D) and cardiovascular diseases [5, 6]. Although the aetiology of PCOS remains unclear, most researchers regard PCOS as multifactorial and suggest that genetic factors play a pivotal role in its development and maintenance [7, 8]. Many studies have reported gene expression profiles based on tissues (i.e. theca cells [9], ovaries [10, 11], oocytes [12] and cumulus cells [13]) from controls and patients with PCOS. Genes associated with PCOS are involved in the insulin receptor signalling pathway, steroid biosynthesis and regulation of gonadotropin secretion [14]. However, the mechanism by which these genes are regulated has not been thoroughly elucidated.

Long noncoding RNAs (lncRNAs) are defined as noncoding RNAs with greater than 200 base pairs [15]. LncRNAs were previously regarded as transcriptional ‘noise’ without biological functions [16]; however, increasing lines of evidence indicate that lncRNAs play key roles in normal development and diseases [17]. To date, several reports have demonstrated that lncRNAs may function in PCOS-related diseases, including T2D, obesity and cardiac diseases. For instance, a β-cell-specific lncRNA (*HI-LNC25*) is dysregulated in T2D by down-regulating the mRNA expression levels of *GLIS3* (Kruppel-like zinc finger transcription factor) [18].

In our previous research [19], we used microarrays [Agilent human lncRNA+mRNA Array v2.0 (4 × 180 K format)] to describe lncRNA profiles in cumulus cells isolated from 10 patients (five patients with PCOS and five normal women). A total of 623 lncRNAs were differentially expressed in PCOS and may contribute to its occurrence [19].Among these lncRNAs, Prader-Willi region nonprotein coding RNA 2 (*PWRN2*) (transcript ID: ENST00000567246.1), which is expressed in the testes and is up-regulated after meiosis during spermatogenesis [20], was found to be up-regulated (3.11-fold) in the cumulus cells of patients with PCOS. Oocyte nuclear maturation has two meiosis resumption processes at the MI (the first meiosis resumption) and MII (the second meiosis resumption) stages. Hence, *PWRN2* may be associated with oocyte nuclear maturation in PCOS. In addition, abnormal folliculogenesis is regarded as a common characteristic of PCOS although its clinical and biochemical signs are typically heterogeneous [21, 22]. Thus, studying the abnormal regulatory mechanisms in oocyte development of PCOS is important.

Increasing lines of evidence suggest that lncRNAs function as miRNA sponges or competing endogenous RNAs (ceRNAs) to reduce the availability of miRNAs for mRNA target binding [23, 24]. In the present study, we confirmed the potential roles of *PWRN2* in oocyte nuclear maturation of PCOS. We then constructed a *PWRN2*- mediated ceRNA network by analysing three microarray datasets (lncRNA+mRNA microarray in PCOS cumulus cells [19], miRNAs microarray in PCOS cumulus cells [25] and lncRNA+mRNA microarray in KGN/shPWRN2 in this study) to investigate the mechanism of *PWRN2*. Results revealed the potential roles of ceRNA in oocyte maturation in PCOS. This work highlights a novel mechanism of oocyte nuclear maturation in PCOS and provides new targets for PCOS treatment.

## Methods

### Patients and IVF treatment

The inclusion criteria for the recruited patients (PCOS and normal) and the methods for collecting CCs were based on our previous reports [19, 26]. This study was approved by the Institutional Ethical Review Board of Tongji University School of Medicine. Sixty participants (30 patients with PCOS and 30 normal) who were referred to our centre for IVF were included in this study after obtaining written informed consent. All patients had no history of taking drugs that affect glucose and lipid metabolism and did not have any known medical conditions or diseases, such as Cushing’s syndrome, congenital adrenal hyperplasia, androgen-secreting tumours and endometriosis. Patients with PCOS were diagnosed according to the revised Rotterdam European Society of Human Reproduction and Embryology/American Society for Reproductive Medicine Criteria [3]. The patients were required to present at least two of the following criteria: chronic oligo-ovulation or anovulation, androgen excess and polycystic ovaries. The inclusion criteria for the recruited patients in this study were as follows: age < 36 years, BMI ranging between 20 and 26 kg/m^2^, basal serum LH/FSH more than 2.0, serum testosterone more than 0.5 ng/mL, antral follicle count ranging between 18 and 35 and number of obtained oocytes ranging between 12 and 28 per cycle. Control patients had regular menstrual cycles, normal ovary sonographs and normal ovulation with bilateral tube occlusion, were nondiabetic and showed no clinical signs of hyperandrogenism and anovulation. The clinical characteristics of control patients and those with PCOS are summarised in Table 1.

**Table 1:** Clinical characteristics of patients

	Normal (n = 30)	PCOS (n = 30)	*P*-value
Age (years)	33.6 ± 2.2	32.6 ± 3.1	NS
BMI (kg/m^2^)	21.4 ± 1.6	21.6 ± 1.5	NS
FSH (mIU/ml)	6.51 ± 1.2	5.41 ± 1.3	< 0.05
LH (mIU/ml)	4.30 ± 1.4	11.91 ± 2.6	< 0.001
Basal LH/FSH	0.65 ± 0.2	2.52 ± 0.61	< 0.001
E2 (pg/ml)	38.5 ± 4.3	41.3 ± 9.5	NS
Testosterone (ng/ml)	0.12 ± 0.05	0.68 ± 0.05	< 0.001
Progesterone (ng/ml)	0.55 ± 0.22	0.70 ± 0.23	NS
Antral follicle count	10.2 ± 1.1	24.1 ± 3.7	< 0.001
Oocytes obtained	8.5 ± 3.0	17.8 ± 5.2	< 0.001
No of MII oocytes	6.3 ± 1.8	14.5 ± 4.6	< 0.001

Data are the mean ± SEM, *NS* not statistically significant

*BMI* body mass index, *E2* oestradiol, *FSH* follicle-stimulating hormone, *LH* luteotropic hormone, *PCOS* polycystic ovary syndrome

Patients in both groups received an agonist protocol as described previously [27]. All patients received the GnRH agonist triptorelin acetate (0.05 mg/day, Diphereline; Ipsen Pharma Biotech, Paris, France) subcutaneously starting at the mid-luteal phase. Once adequate pituitary down-regulation was confirmed [serum LH levels < 3.0 ng/mL and serum estradiol (E_2_) levels < 30 pg/mL], the patients received recombinant FSH (150–187.5 IU; Gonal-f, Follitropin Alfa, Serono) subcutaneously for COS. When two or more follicles were at least 18 mm in diameter and the serum E_2_ levels were at least 300 pg/mL per dominant follicle, all patients received 250 μg of hCG (Profasi, Serono).

### Retrieval of cumulus cells

Collection of CCs and assessment of oocytes were conducted as previously described [27, 28]. Cumulus-oocyte complex (COC) retrieval was performed by vaginal puncture under ultrasound echo-guidance 36 h after hCG administration. After COC retrieval, a portion of CCs surrounding a single oocyte was removed using a sharp needle. For RNA extraction, the cumulus cells were lysed in 80 μL of lysis buffer (mirVana miRNA Isolation Kit; Ambion, Austin, TX, USA) and stored at − 80 °C. For vector transfection and luciferase activity assay, the cumulus cells were firstly digested with trypsin and then cultured directly. Oocytes were further inseminated by ICSI and cultured in sequential media of SAGE (CooperSurgical, Leisegang Medical, Berlin) individually in 20 μL of droplets covered with mineral oil. The embryos were transferred or vitrified on day 3, and the other embryos were cultured to blastula stage on days 5–6.

### Assessment of oocyte and division of the groups of cumulus cells

The morphological characteristics of the oocytes were individually recorded. The oocytes were denudated to assess the maturation stage before ICSI. Few of germinal vesicle (GV)-stage COCs (12 in patients with PCOS and only 3 in normal patients) were retrieved. We classified the COCs into two categories based on nuclear status: (i) MI/GV group: immature MI oocytes exhibiting no polar bodies (PB) or immature oocytes at the GV stage, and (ii) MII group: mature MII oocytes that extruded a clearly visible PB. The corresponding cumulus cells were divided into CC_MI/GV_ and CC_MII_ groups. Each group had ≥ three replicates. Each subgroup, containing at least 15 cumulus cells, represented a biological replicate. Each CC_MI/GV_ subgroup has one CC_GV_.

### RNA extraction

Total RNA was isolated using a Qiagen RNeasy Mini Kit (Qiagen, Hilder, Germany) according to the manufacturer’s instructions. This RNA isolation kit significantly reduced contamination from genomic DNA and proteins. The purity and concentration of RNA were determined from OD260/280 readings using a spectrophotometer (NanoDrop ND-1000). RNA integrity was determined using 1% formaldehyde denaturing gel electrophoresis.

### qRT-PCR

The expression levels of *PWRN2* in the CC_MI/GV_ and CC_MII_ groups of normal patients and those with PCOS were tested by qRT-PCR analysis to evaluate the correlation of changes in *PWRN2* with oocyte maturation. The potential ceRNA network was constructed to investigate the action mechanism of *PWRN2*.The co-expression characteristics of the candidate genes of the ceRNA network were also tested in the CCs corresponding to oocytes at different nuclear maturity stages (MI/GV and MII) of patients with PCOS through qRT-PCR analysis.

Total RNA was reverse transcribed into cDNA by using a miScript Reverse Transcription Kit (Qiagen). qRT-PCR analysis was performed using SYBR green assay (Takara Bio, Inc., Dalian, China) according to the manufacturer’s protocols. PCR was performed in a total reaction volume of 20 μL containing 10 μL of 2× QuantiTest SYBR Green PCR Master Mix, 1 μL of cDNA template, 1 μL of each primer and RNase-free water. The primers used in this study are listed in Additional file 1: Table S1. All reactions were performed using the ABI PRISM 7300 system. The amplification conditions were as follows: 10 min at 98 °C; 40 cycles of 15 s at 95 °C, 1 min at 60 °C; and a final extension for 5 min at 72 °C. Amplification efficiency was evaluated by standard curve analysis. *PWRN2* and mRNA expression data were normalised to those of *GAPDH*. The miRNA expression data were normalised to U6. Each set of qRT-PCR reactions was repeated at least three times, and fold change in the expression of each gene was analysed by the ΔΔCt method [29].

### KGN cell culture

*PWRN2*-regulated genes were identified using RNA interference technology to inhibit the expression of *PWRN2* in KGN cell lines and eliminate differences in the genetic backgrounds of different patients with PCOS. KGN (RCB1154; RIKEN, Wako, Japan) is a steroidogenic human ovarian granulosa tumour cell line. KGN cells were cultured in 1:1 Dulbecco’s modified Eagle’s medium and Ham’s F-12 medium (DMEM/ F12; Nissui Pharmaceutical, Tokyo, Japan) supplemented with 10% foetal bovine serum (FBS) and antibiotics (100 IU/mL penicillin and 100 μg/mL streptomycin). On the day before lentivirus transfection, KGN cells were placed into the medium without serum and incubated overnight.

### Construction of lentivirus shRNA and cell transfections

Lentivirus shRNA construction and cell transfection were conducted using previously described methods [30, 31]. We selected three target sequences to construct lentiviral shRNAs (LV-*PWRN2*-homo-502, LV-*PWRN2*-homo-1574 and LV-*PWRN2*-homo-1261) and included a negative control (LV-NC) (Table 2). The target sequences were used to design two complementary oligonucleotides, which were synthesised and cloned into pGLV3/H1/GFP + Puro Vector (GenePharma, China). The positive purified lentiviral shRNA-expressing plasmids were transfected with packaging plasmids into 293 T cells for lentivirus generation (GenePharma, China). The vectors described above were used to infect KGN cells. Stable KGN cell lines were selected using 3 μg/mL bulk puromycin-resistance culture (puromycin, Sigma, St Louis, MO, USA) for 5 days. Afterwards, the cells were examined microscopically for lentiviral GFP expression. The expression levels of *PWRN2* in KGN/sh*PWRN2* cells and the corresponding negative-control KGN cells were tested by qRT-PCR to validate the effects of RNA interference.

**Table 2:** Target sequences of lentiviral shRNAs for interfering *PWRN2*

LV-sh*PWRN2*	Site	Target sequences
PWRN2-homo-502	502–522	5’-GCCATTCGGTTACCATCTACT-3’
PWRN2-homo-1574	1574–1594	5’-GCAAAGGAATTACCGTTTACA-3’
PWRN2-homo-1261	1261–1281	5’-GGCAGAAAGCAATGAAGAAGA-3’
NC	Nonsense	5’-TTCTCCGAACGTGTCACGT-3’

### Microarray hybridisation and data analysis

For microarray analysis, three KGN/shPWRN2 cell lines with down-regulated (FC < 0.5) *PWRN2* mRNA levels were selected as shPWRN2 groups. The corresponding three KGN cell lines with negative control vectors were used as the control groups. The methods for RNA labelling, array hybridisation and data analysis were described in our previous report [19]. The purified RNA extracted from KGN/sh*PWRN2* samples or normal KGN cells was amplified and transcribed into fluorescent cDNA for hybridisation to the Agilent human lncRNA+ mRNA Array v4.0 (4 × 180 K format) with each array containing probes interrogating approximately 41,000 human lncRNAs and approximately 34,000 human mRNAs. The lncRNA+mRNA array data were analysed to summarise, normalise and assess the quality of the data using GeneSpring software V11.5 (Agilent). To select differentially expressed genes, we used threshold values of ≥2.0- and ≤ − 2.0-fold changes and a Benjamini–Hochberg corrected *P* value of 0.05. The data were log2 transformed and median centred by genes using the Adjust Data function of the CLUSTER 3.0 software. The data were further analysed using hierarchical clustering with average linkages. Finally, we visualised the tree using Java TreeView (Stanford University School of Medicine, Stanford, CA, USA). Microarray hybridisation and data analysis were performed by CapitalBio Corporation, Beijing, P. R. China.

### Gene ontology (GO) and KEGG pathway analyses

GO analysis was performed to describe genes and gene product attributes in any organism (http://www.geneontology.org). This ontology covers three domains: biological processes, cellular components and molecular functions. The *P* value denotes the significance of the GO term enrichment among differentially expressed genes (P value ≤0.05 is recommended). For pathway analysis, we used the free web-based Molecular Annotation System 3.0 (MAS 3.0; http://bioinfo.capitalbio.com/mas3/), which integrates three different open-source pathway resources: KEGG, BioCarta and GenMAPP. The significantly altered pathways were selected using the threshold of the P value and FDR (corrected P value) < 0.05 derived from the hypergenomic test. GO and KEGG pathway analyses were performed by CapitalBio Corporation, Beijing, P. R. China.

### Construction of the *PWRN2*-mediated ceRNA network based on microarray data

A potential *PWRN2*-mediated ceRNA network was constructed based on three microarray datasets to explain whether *PWRN2* functions as miRNA sponges or ceRNAs. The putative miRNAs and mRNAs included in the construction of the *PWRN2*-mediated ceRNA network are as follows: (i) miRNA selection: miRNAs were predicted to possess *PWRN2* binding sites by using miRanda v3.3a software and were compared with previous miRNA microarray data of PCOS; putative miRNAs with MREs in *PWRN2* and identified from previous microarray data of PCOS were selected; and (ii) mRNA selection: the target genes of the miRNAs were predicted using miRbase (http://www.mirbase.org/) and compared with the lncRNA+mRNA microarray data from KGN cells with *PWRN* interference. The selected mRNAs met two criteria: 1) differentially expressed mRNAs identified from our lncRNA+mRNA microarray data; and 2) putative target genes of miRNAs with MREs in *PWRN2*.

### Luciferase reporter constructs and luciferase activity assay

The direct interaction between the candidate genes of *PWRN2*-mediated ceRNA network was evaluated by luciferase activity assay. A luciferase reporter vector (pmirGLO Dual-Luciferase miRNA Target Expression Vector; Promega) was used for luciferase constructs. *PWRN2* and the 3’UTR of *TMEM120B* were cloned by RT-PCR. *PWRN2*-WT, *PWRN2-*mutant and *TMEM120B-* 3′UTR (WT and mutant) were constructed as previously reported [26, 32]. Cumulus cells of patients with PCOS were digested with 0.25% trypsin for 5 min. The digestion was inhibited by adding 2.5 mL of DMEM: F12 containing 10% FBS and by incubating at room temperature for 5–10 min. The cumulus cells were pooled in a sterile 15 mL conical tube on ice, centrifuged at 200 g for 10 min, washed once with sterile saline, centrifuged again and resuspended in 3 mL of culture media. The number of viable cells was determined by trypan blue exclusion and ranged between 35 and 45%. The cumulus cells were plated onto 24-well plates and allowed to grow for ~ 24 h before transfection. The constructed reporter vectors (300 ng) were transfected into cells together with the miRNA and control mimics (100 nM) in Lipofectamine 2000 (2 μL). The cells were lysed after ~ 24 h of transfection. Luciferase activity was assayed using the Dual-Luciferase Reporter Assay System (Promega). Firefly luciferase activities were normalised to Renilla luciferase activities. The experiments were performed independently in triplicate.

### Statistical analysis

All statistical analyses were performed using SPSS 17.0 software (SPSS Inc.) unless otherwise noted. Differences in the expression levels of the candidate genes of the *PWRN2*–mediated ceRNA network in cumulus cell samples were evaluated by two-tailed t-test. Differences were considered statistically significant at *P* < 0.05.

## Results

### Expression levels of *PWRN2* in cumulus cells according to oocyte nuclear maturity in normal patients and those with PCOS

**Fig. 1:**
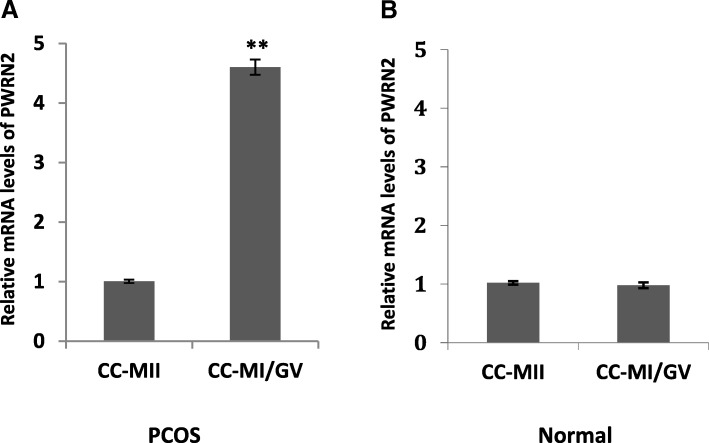
Transcript levels of *PWRN2* according to oocyte nuclear maturity in PCOS (*n* = 30) and normal patients (*n* = 30). Expression levels of *PWRN2* in cumulus cells of different oocyte nuclear maturity stages (MI/GV stage and MII stage) of patients with PCOS (**a**) and normal patients (**b**). The signal intensity for *PWRN2* is shown on the y-axis in arbitrary units determined by qRT-PCR analysis with *GAPDH* as an endogenous reference. * indicates a significant difference in gene expression between CC categories (** *P* < 0.01, * *P* < 0.05). The results are presented as means ± SEM. CC_MI/GV_: cumulus cells from oocytes at the MI or GV stage; CC_MII_: cumulus cells from oocytes at the MII stage

The transcript levels of *PWRN2* were detected in the CC_MI/GV_ and CC_MII_ groups to evaluate the involvement of *PWRN2* in the oocyte nuclear maturity in patients with PCOS (*n* = 30) and normal patients (*n* = 30). For PCOS, the mean transcript levels of *PWRN2* in the CC_MII_ group was significantly down-regulated by 4.55–fold compared with that in the CC_MI/GV_ group (1.006 ± 0.028 versus 4.602 ± 0.128; Fig. 1A). In normal patients, the expression levels of *PWRN2* exhibited no significant differences between the CC_MII_ and CC_MI/GV_ groups (1.02 ± 0.029 versus 0.98 ± 0.049; Fig. 1B). These results indicated that the *PWRN2* expression pattern during oocyte maturation differed between normal patients and those with PCOS.

### Expression profiles of lncRNAs and mRNAs in KGN/sh*PWRN2* cell lines

**Fig. 2:**
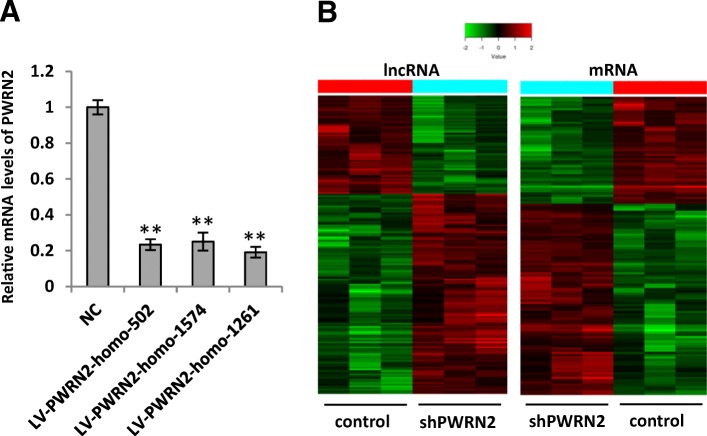
Expression profiles of lncRNAs and mRNAs in KGN/shPWRN2 cell lines. **a** Relative expression of *PWRN2* mRNA was examined in KGN cells infected with different lentiviral shRNAs (LV-*PWRN2*-homo-502, LV-*PWRN2*-homo-1574, LV-*PWRN2*-homo-1261, or LV negative control) using qRT-PCR analysis with *GAPDH* as an endogenous reference. The KGN cells treated with different lentiviral shRNAs are shown on the x-axis, and the relative change of *PWRN2*/*GAPDH* is shown on the y-axis. Each set of qRT-PCR reactions was repeated at least three times. The results are presented as mean ± SEM. ** indicates *P* < 0.01. **b** Cluster of lncRNAs and mRNAs that were dyexpressed in KGN/sh*PWRN2* cells. From this lncRNA+mRNA microarray data, 176 lncRNAs changed significantly in KGN/sh*PWRN2* cells compared with those in the control cell lines. Among these lncRNAs, 118 were up-regulated, whereas 58 lncRNAs were down-regulated; and 131 mRNAs changed significantly in the KGN/sh*PWRN2* cell lines. Among these, 84 mRNAs were up-regulated, and 47 mRNAs were down-regulated. The supervised hierarchical clustering of genes overexpressed in KGN/sh*PWRN2* cells is shown. Distinct signatures were observed in the KGN/sh*PWRN2* cells. The value of each gene was adjusted with a median-centring algorithm on a log scale; the colours indicate the relative gene expression in the red-green heat map. Pure black indicates 0.00 and represents no change in the median gene expression levels in all samples. Pure green indicates − 2.00 and represents lower expression. Pure red indicates + 2.00 and relatively higher expression

To avoid the different genetic backgrounds of paitents with PCOS, we used KGN cell lines to identify *PWRN2*-regulated genes by RNA interference. Lentiviral shRNAs were constructed to inhibit *PWRN2* expression in KGN cells. The results of qRT-PCR analysis indicated that the relative expression levels of *PWRN2* mRNA were 0.23, 0.25 and 0.19 in KGN cells treated with LV-*PWRN2*-homo-502, LV-*PWRN2*-homo-1574 and LV-*PWRN2*-homo-1261, respectively, compared with those in the LV-NC group (Fig. 2A). These results indicated that the three lentiviral shRNAs effectively inhibited *PWRN2* replication in KGN cells.

The lncRNA and mRNA expression profiles of KGN/sh*PWRN2* cells infected with LV-*PWRN2*-homo-502, LV-*PWRN2*-homo-1574 or LV-*PWRN2*-homo-1261 were examined by microarrays and compared with those of the three KGN cell lines infected with control LV-NC. The raw microarray data were deposited in NCBI’s Gene Expression Omnibus (GEO; http://www.ncbi.nlm.nih.gov/geo/) and can be accessed through the GEO series accession number GSE97772. The microarray analysis revealed thousands of lncRNAs and mRNAs expressed in KGN/sh*PWRN2*.

The expression of 176 lncRNAs significantly changed in KGN/sh*PWRN2* cells relative to the control KGN cell lines. A total of 118 lncRNAs were up-regulated, whereas 58 lncRNAs were down-regulated. A total of 131 mRNAs significantly changed in the KGN/sh*PWRN2* cell lines. About 84 mRNAs were up-regulated and 47 mRNAs were down-regulated (fold change ≥2.0, *P* < 0.05). Clustering analysis of the microarrays based on the differentially expressed lncRNAs or mRNAs clustered the KGN/sh*PWRN2* groups and the control groups (Fig. 2B).

The top 20 up and down-regulated lncRNAs and mRNAs are listed in Tables 3 and 4, respectively. From the microarray data, the expression level of *PWRN2* was down-regulated (FC = − 2.526) in the KGN/sh*PWRN2* groups. The results were in concordance with the qRT-PCR data of *PWRN2* expression levels in the KGN/sh*PWRN2* groups, indicating the reliability of lncRNA+mRNA microarray data.

**Table 3:** The top 20 up-regulated and down-regulated lncRNAs in KGN/shPWRN2 cells

Up-regulated lncRNAs		Down-regulated lncRNAs	
lncRNA ID (or known lncRNA name)	Fold-change	lncRNA ID (or known lncRNA name)	Fold-change
XR_110904.1	5.369	TCONS_00001136	4.033
XR_430233.1	4.498	ENST00000557828.1	3.875
ENST00000584157.1	4.485	RNA143578	3.422
RNA147334|p0438_imsncRNA843	4.185	RNA143598	3.292
uc003qmi.3	4.118	RNA146915|p0019_imsncRNA52	3.097
ENST00000543573.1	4.072	TCONS_00019731	3.092
TCONS_00012676	3.934	TCONS_00025502	3.088
ENST00000500823.2(GS1-24F4.2)	3.730	HIT000271588	3.068
ENST00000428520.2(LINC00710)	3.440	ENST00000567517.1(LINC00515)	3.029
ENST00000502766.2	3.253	RNA147684|p0788_imsncRNA576	2.963
ENST00000608299.1	3.252	HIT000468529	2.883
ENST00000607195.1	3.047	TCONS_00019573	2.882
XR_245201.2	3.035	ENST00000452741.1	2.725
uc.311-	3.034	RNA143600	2.707
TCONS_00009112	3.013	TCONS_00010743	2.638
ENST00000570809.1	2.997	TCONS_00010435	2.628
uc.211-	2.975	ENST00000489626.1	2.551
ENST00000602984.1(MIR137HG)	2.960	uc022caj.1	2.548
TCONS_00000870	2.943	ENST00000566245.1(PWNR2)	2.526
ENST00000513586.1	2.928	ENST00000439960.1	2.445

**Table 4:** The top 20 up-regulated and down-regulated mRNAs in KGN/shPWRN2 cells

Up-regulated mRNAs			Down-regulated mRNAs		
GeneSymbol	Genbank Accession	Fold-change	GeneSymbol	Genbank Accession	Fold-change
CACTIN	NM_021231	5.976	FTH1	NM_002032	7.358
ND3	HV963894	4.613	FAM154A	NM_153707	3.460
NUB1	NM_016118	4.417	MOP-1	AB014771	3.421
CCDC6	NM_005436	3.410	MARVELD2	AK055094	3.176
MLF1	NM_022443	3.227	RPL19	NM_000981	3.175
GPI	NM_000175	3.108	CCNG2	NM_004354	3.162
ADAM11	NM_002390	3.066	TMEM120B	NM_001080825	3.090
SYNGR4	NM_012451	2.916	SNX22	XM_005254677	3.058
TMEM234	NM_019118	2.855	RGS4	BC000737	3.051
PHIP	NM_017934	2.711	FBRSL1	NM_001142641	2.985
HIF3A	XR_243952	2.685	MB21D1	NM_138441	2.961
NIP7	NM_016101	2.644	ND4	AK097322	2.779
C11orf45	NM_145013	2.636	FMNL1	NM_005892	2.754
VWA1	NM_022834	2.565	NHS	NM_198270	2.753
ZSCAN32	NM_017810	2.562	SEMA6B	NM_032108	2.752
RPPH1	CA413366	2.553	RPL32	NM_001007074	2.729
EPHA4	NM_004438	2.551	TLE6	NM_024760	2.564
CCDC102B	NM_024781	2.403	GRIP2	NM_001080423	2.545
NOS1AP	NM_014697	2.378	LOC100287036	NM_001242885	2.454
CEP70	NM_024491	2.377	RPS2	NM_002952	2.372

### GO and KEGG pathway analyses

The genes regulated by *PWRN2* were selected to identify the associated signalling pathways and biological functions. Regarding GO annotation, the biological processes, cellular components and molecular function associated with the target genes are presented in Additional file 2: Table S2. The GO analysis showed that the most significantly altered biological processes were nuclear-transcribed mRNA catabolic process, RNA catabolic process, SRP-dependent co-translational protein targeting to the membrane, protein targeting to the ER and others. Moreover, the KEGG pathway analysis demonstrated that the most significantly altered pathways were mRNA surveillance pathways, carbon metabolism, nitrogen metabolism and axon guidance (Additional file 3: Table S3). These results indicated that *PWRN2*-regulated genes were mostly involved in different metabolic pathways, thereby confirming that PCOS is a complex metabolic disease.

### Construction of a *PWRN2*-regulated ceRNA network based on three microarray datasets

Recent studies reported that lncRNAs have been reported to function as competing endogenous RNAs [33] by mopping up miRNAs, thereby regulating their function [34]. Given that lncRNAs can interact with miRNAs through their MREs within a ceRNA network [23], we searched for putative miRNAs with MREs in *PWRN2* by using miRanda software. A total of 69 miRNAs were predicted to possess *PWRN2* binding sites. The potential target mRNAs of these 69 miRNAs were also predicted (Additional file 4: Table S4).

We further compared the 69 potential *PWRN2*-regulated miRNAs with the differentially expressed miRNAs (21 up-regulated and 38 down-regulated) in the cumulus cells of patients with PCOS [25]. Two miRNAs (miR-92b-3p and miR-365b-5p) were found. *PWRN2* was up-regulated in PCOS cumulus cells in our previous report [19], and the miRNAs (miR-92b-3p and miR-365b-5p) that possess *PWRN2* binding sites were down-regulated [25]. Hence, *PWRN2* may function as ceRNA to regulate miRNAs (miR-92b-3p and miR-365b-5p) in cumulus cells in PCOS.

**Fig. 3:**
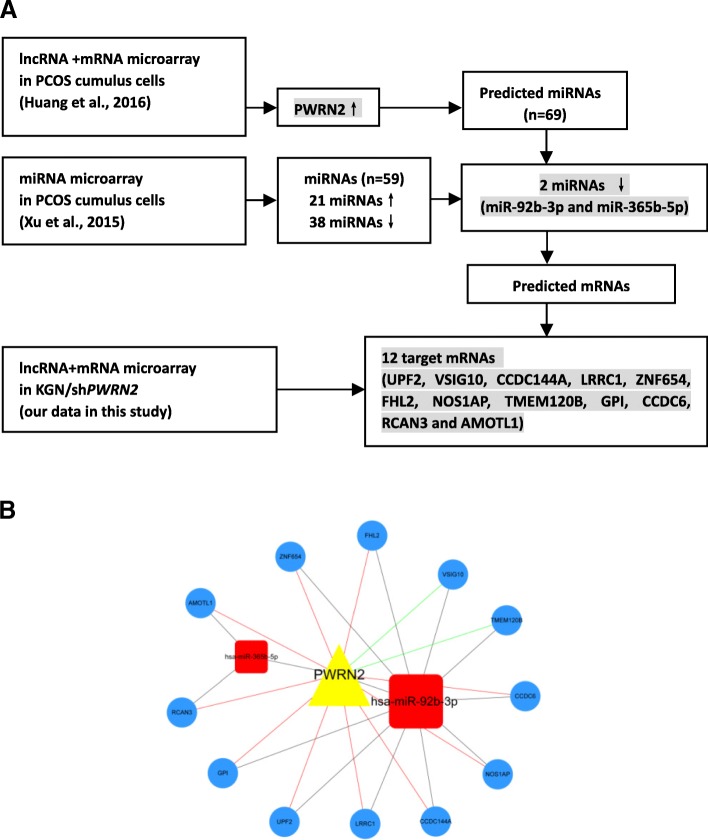
Construction of a *PWRN2*-mediated ceRNA network based on microarray datasets. **a** Flow chart of building a *PWRN2*-mediated ceRNA network based on three microarray datasets: lncRNA+mRNA microarray in PCOS cumulus cells, miRNA microarray in PCOS cumulus cells and lncRNA+mRNA microarray in KGN/sh*PWRN2*. **b** *PWRN2*-mediated ceRNA network. The ceRNA network is based on lncRNA/miRNA, lncRNA/mRNA and miRNA/mRNA interactions. In this network, black edges represent sequence matching, green edges represent positive correlations and red edges represent negative correlations

By comparing the potential target genes of miR-92b-3p and miR-365b-5p with the *PWRN2*-regulated mRNAs identified by KGN/shPWRN2 microarray, we found 12 mRNAs, including *UPF2*, *VSIG10*, *CCDC144A*, *LRRC1*, *ZNF654*, *FHL2*, *NOS1AP*, *TMEM120B*, *GPI*, *CCDC6*, *RCAN3* and *AMOTL1*. Thus, a credible *PWRN2*-miRNA-mRNA ceRNA network was constructed using the combined analyses of the three microarrays (lncRNA+mRNA microarray in PCOS, miRNA microarray in PCOS and lncRNA+mRNA microarray in KGN/sh*PWRN2*; Figs. 3A, B).

According to our previous analysis of lncRNA and mRNA microarray data, *PWRN2* and *TMEM120B* were over-expressed in PCOS cumulus cells [19]. In this study, the expression level of *TMEM120B* was down-regulated in KGN/sh*PWRN2* cells. The results indicated that the expression trends of *PWRN2* and *TMEM120B* were consistent with PCOS. Moreover, miR-92b, which was predicted to process the MREs in *PWRN2* and to have a binding site in the 3′-UTR of *TMEM120B*, was down-regulated in PCOS cumulus cells (Table 5). Therefore, we regarded the *PWRN2*-miR-92b-*TMEM120B* ceRNA network as more reasonable and credible.

**Table 5:** The expression levels of *PWRN2*, miR-92b, and *TMEM120B* in three microarray data

Microarray data	*PWRN2*	miR-92b	*TMEM120B*
lncRNA+mRNA array in PCOS cumulus cells (Huang et al., 2016)	Up-regulated FC = 3.11	no	Up-regulated FC = 2.02
miRNA array in PCOS cumulus cells (Xu et al., 2015)	no	Down-regualted FC = −2.27	no
lncRNA+mRNA array in KGN/shPWRN2 array (this study)	Down-regulated FC = −2.52	no	Down-regulated FC = −3.09

### Co-expression characteristics of genes of the *PWRN2*-miR-92b-3p-*TMEM120B* ceRNA network in PCOS

**Fig. 4:**
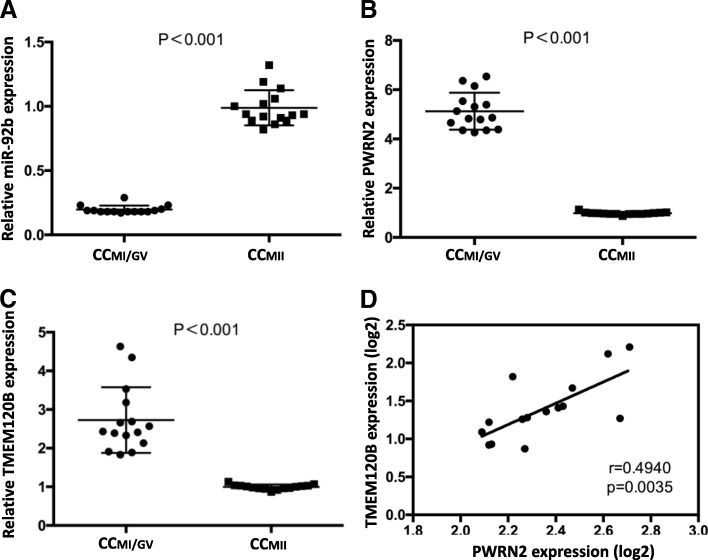
Transcripts levels of the candidate genes of *PWRN2*-miR-92b-3p-*TMEM120B* ceRNA network according to oocyte nuclear maturity in patients with PCOS (*n* = 30). The transcript levels of miR-92b-3p (**a**), *PWRN2* (**b**) and *TMEM120B* (**c**) were detected by qRT-PCR. **d** Relationship between the expression levels of *PWRN2* and *TMEM120B*. The signal intensity for the genes is shown on the y-axis in arbitrary units determined by qRT-PCR analysis. *GAPDH* was used as internal control for *PWRN2* and *TMEM120B*, while U6 was used as internal control for miR-92b-3p. * indicates a significant difference in gene expression between CC categories (** *P* < 0.01, * *P* < 0.05). The results were presented as means ± SEM. CC_MI/GV_: cumulus cells from oocyte at the MI or GV stage; CC_MII_: cumulus cells from oocyte at the MII stage

The expression of the three candidate genes (*PWRN2*, miR-92b-3p and *TMEM120B*) in cumulus cell according to oocyte nuclear maturity (CC_MI/GV_ and CC_MII_) in patients with PCOS (*n* = 30) were detected by qRT-PCR to validate the potential ceRNA network. Based on comparison between CC_MII_ and CC_MI/GV_ groups, the mean transcript level of miR-92b-3p in CC_MII_ group was significantly 4.95–fold higher than that in the CC_MI/GV_ group (0.99 ± 0.14 versus 0.20 ± 0.03) (Fig. 4A); by contrast, the expression levels of the other genes (*PWRN2* and *TMEM120B*) significantly decreased in the CC_MII_ samples, with 5.23– and 2.73–fold decrease observed for *PWRN2* (0.98 ± 0.05 versus 5.13 ± 0.75) (Fig. 4B) and *TMEM120B* (1.0 ± 0.06 versus 2.73 ± 0.85) (Fig. 4C), respectively. A significant positive correlation was observed between *PWRN2* and *TMEM120B* (Fig. 4D). The co-expression characteristics observed were in accordance with the ceRNA hypothesis.

### Confirmation of the direct interactions of the candidate genes of the *PWRN2*-miR-92b-3p-*TMEM120B* ceRNA network

**Fig. 5:**
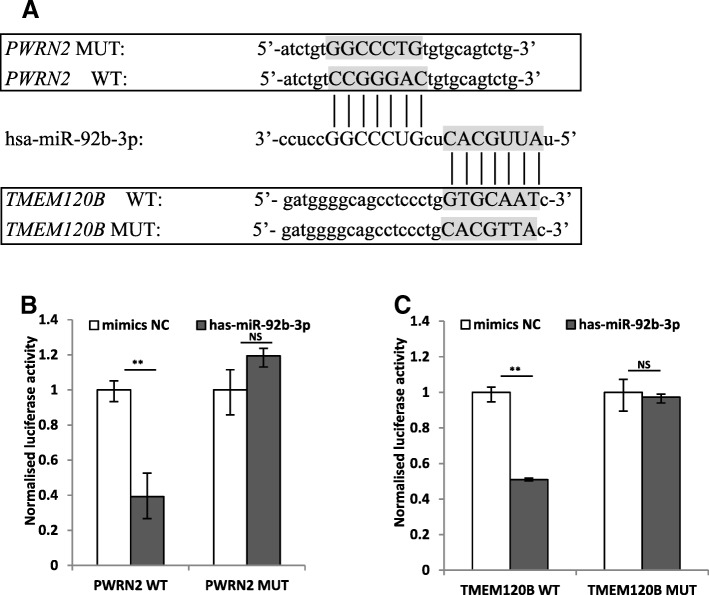
Validation miR-92b-3p directly binds to *PWRN2* and the direct interaction between miR-92b-3p and its predictive target gene (*TMEM120B*) by the dual luciferase activity assay. **a** Interaction regions of *PWRN2*/miR-92b-3p and miR-92b-3p/*TMEM120B* predicted by miRanda software. MiR-92b-3p, which is predicted to process the MREs in *PWRN2*, also has a binding site in the 3′-UTR of *TMEM120B*. **b** The relative luciferase activity was assayed following co-transfection of miR-92b-3p mimics with the constructs encoding the wild-type or mutant *PWRN2* cDNA into the cumulus cells. **c** The relative luciferase activity was assayed following co-transfection of miR-92b-3p mimics with the constructs encoding the wild-type or mutant miR-92b-3p binding site of *TMEM120B* 3′-UTR into the cumulus cells. The constructed reporter vectors (WT or MUT) are shown on the x-axis, and the y-axis represents the normalised luciferase activity (firefly luciferase activities were normalised to renilla luciferase activities). The experiments were performed independently in triplicate. * indicates *P* < 0.05, ** indicates *P* < 0.01, NS indicates no significant difference. The results are presented as means ±S.E.M

According to the prediction results, *PWRN2* has one putative miR-92b-3p binding site. We recombined the *PWRN2* cDNA (*PWRN2*-WT) and mutational cDNA (*PWRN2*-MUT) with the presumed miR-92b-3p deleted recognition sequences downstream the luciferase reporter gene (Fig. 5A). The vectors were transfected in PCOS cumulus cells along with the corresponding miRNA mimics. After miR-92b-3p mimics transfection, luciferase activity was reduced by 60% compared with the control miRNA (Fig. 5B). Hence, miR-92b-3p directly binds to *PWRN2*.

To determine whether *TMEM120B* is a direct target of miR-92b-3p, we constructed luciferase reporter constructs by cloning the DNA segment-encoding part of the WT or mutant (which cannot bind miR-92b-3p) 3′UTR of *TMEM120B* mRNA downstream of the Renilla luciferase gene (Fig. 5A) and transfected them with miR-92b-3p mimics into the PCOS cumulus cells. As shown in Fig. 5C, the relative luciferase activity significantly decreased in cumulus cells co-transfected with the *TMEM120B* WT constructs and miR-92b-3p mimics but not in cells co-transfected with the *TMEM120B* mutant constructs and miR-92b-3p mimics. Thus, miR-92b-3p suppressed the expression of *TMEM120B* in the cumulus cells by directly binding to the 3′UTR of *TMEM120B* mRNA.

## Discussion

*PWRN2* was up-regulated in PCOS cumulus cells and could be involved in oocyte development [19]. In the present study, we proved that *PWRN2* is associated with oocyte nuclear maturation in patients with PCOS, in contrast to that in normal patients. Hence, *PWRN2* plays important roles during the oocyte development in PCOS.

To elucidate the roles of *PWRN2* in oocyte development, we identified *PWRN2*-regulated genes in KGN cells by using RNA interference technology. A total of 176 lncRNAs and 131 mRNAs were identified and determined to be regulated by *PWRN2*.The GO and KEGG pathway analyses of these DEGs showed that the differentially expressed genes were mostly involved in various catabolic processes (e.g. nuclear-transcribed mRNA and RNA catabolic processes) and metabolism pathways (e.g. carbon and nitrogen metabolism). These results indicated that *PWRN2* may play an important role in metabolic processes. Metabolic abnormalities in cumulus cells in PCOS change the follicular microenvironment during oocyte development and subsequently affect oocyte quality [35, 36]. This deduction is consistent with PCOS because it is a metabolic disease often accompanied by poor oocyte developmental potential.

Although potential target genes of *PWRN2* were identified, the molecular mechanism of *PWRN2* remains largely unknown. Several recent reports provided support for the ceRNA hypothesis, which accounts for the function of a substantial proportion of uncharacterised lncRNAs [37–40]. To examine if *PWRN2* functions as a ceRNA, we developed a new method for elucidating specific lncRNA-mediated ceRNA networks. The most widely used methods for exploring potential lncRNA-miRNA-mRNA networks largely depend on shared MREs that are predicted by miRNA target discovery algorithms [41, 42]. We predicted miRNAs that possess *PWRN2* binding sites and potential miRNA-mRNA pairs. We then obtained *PWRN2*-mediated miRNAs by combining the analyses of predicted miRNAs and miRNA microarray data from previous report [25]. Meanwhile, *PWRN2*-regulated mRNAs were obtained by comparing potential miRNA-target mRNA with the microarray data from KGN/sh*PWRN2* in the present study. Finally, we incorporated a bioinformatic prediction tool to construct a *PWRN2*-miRNA-mRNA ceRNA network. Based on the dual-luciferase activity assay, the ceRNA network is reliable because it is based on datasets from three microarrays.

Furthermore, we investigated *PWRN2* and miR-92b-*TMEM120B* pair and validated their expression levels in cumulus cells according to oocyte nuclear maturity (CC_MI/GV_ and CC_MII_) of patients with PCOS. The results were in accordance with the ceRNA hypothesis. In this regard, *PWRN2* functions as ceRNA to reduce the availability of miR-92b-3p for *TMEM120B* target binding. *TMEM120B* is a fat-specific nuclear envelope transmembrane protein that may play a contributory role in adipogenesis [43]. In PCOS, up-regulated *TMEM120B* will promote adipocyte differentiation/metabolism and induce obesity. Severe obesity has been shown to be associated with a high prevalence of spindle anomalies and non-aligned chromosomes in failed fertilised oocytes [44]. Thus, the ceRNA network of *PWRN2*-miR-92b-*TMEM120B* provides a basis for explaining the poor quality of oocyte in patients with PCOS.

Our study presents limitations. Firstly, we cannot identify large lncRNA-miRNA-mRNA ceRNA networks by combining microarray datasets. Moreover, the molecular roles of the *PWRN2*-miR-92b-*TMEM120B* network in oocyte development in PCOS require further investigation. Secondly, a large number of samples must be analysed to validate the ceRNA network in PCOS. Our future work will further validate the *PWRN2*-miR-92b-*TMEM120B* network and elucidate its role in the pathogenesis of PCOS, especially in oocyte development.

In conclusion, our results proved that lncRNA (*PWRN2*) is associated with oocyte nuclear maturation in PCOS. The constructed *PWRN2*-miR-92b-*TMEM120B* ceRNA network based on three microarray datasets indicated that *PWRN2* functions as ceRNA to reduce the availability of miR-92b-3p for *TMEM120B* target binding during oocyte nuclear maturation in PCOS. This ceRNA network provides new information and helps clarify the metabolic disorder that induces abnormal oocyte development in PCOS.

## Additional files

Additional file 1: The primers used in this study. (XLSX 12 kb)

Additional file 2: GO annotation of the target genes regulated by PWRN2. (XLSX 283 kb)

Additional file 3: KEGG pathway analysis of the target genes regulated by PWRN2. (XLSX 35 kb)

Additional file 4: The potential target mRNAs of putative miRNAs with MREs in PWRN2. (XLSX 97 kb)

## Abbreviations

CCs: Cumulus cells
ceRNA: competing endogenous RNAs
COC: Cumulus-oocyte complex
lncRNA: long non-coding RNAs
PCOS: Polycystic ovary syndrome
*PWRN2*: Prader-Willi region non-protein coding RNA 2
